# Chemotherapy-related subjective cognitive impairment in breast cancer patients in semi-rural South Africa

**DOI:** 10.4102/hsag.v26i0.1605

**Published:** 2021-07-26

**Authors:** Nicholas M. Keetile, Elzbieta Osuch, Antonio G. Lentoor

**Affiliations:** 1 Department of Pharmacology, School of Medicine, Sefako Makgatho Health Sciences University, Pretoria, South Africa; 2 Department of Clinical Psychology, School of Medicine, Sefako Makgatho Health Sciences University, Pretoria, South Africa

**Keywords:** breast cancer, adjuvant chemotherapy, Fact-Cog test, perceived cognitive impairment, chemotherapy-related cognitive impairment (CRCI), chemobrain

## Abstract

**Background:**

Breast cancer is the most commonly diagnosed cancer amongst women worldwide. Whilst current evidence indicates the therapeutic benefits from the use of chemotherapy, self-perceived cognitive difficulties emerged as a frequent occurrence during and after chemotherapy treatment in breast cancer patients.

**Aim:**

The current study sought to investigate self-perceived cognitive impairment in a group of breast cancer patients in semi-rural South Africa.

**Setting:**

The patients were recruited from an outpatient oncology clinic at a semi-rural, tertiary academic hospital in Gauteng, South Africa.

**Methods:**

In a randomised, quantitative, time-based series study, 30 female patients aged 21–60 years (mean age = 50 years) diagnosed with stages II and III breast cancer on CMF (cyclophosphamide, methotrexate, fluorouracil) (*n* = 10) and FAC (fluorouracil, adriamycin, cyclophosphamide) (*n* = 20) chemotherapy regimens, completed the self-reported Functional Assessment of Cancer Therapy-Cognition (Fact-Cog) test as a measure of subjective cognitive functioning at three points during the course of treatment (T_0_, T_1_, T_2_).

**Results:**

The results of the paired sample *t*-tests showed the scores on the Fact-Cog test confirmed significant cognitive decline for both treatment groups from baseline (T_0_) to completion (T_2_) of chemotherapy; CMF group, *t* (9) = 2.91, *p* = 0.017 and the FAC group *t* (19) = 4.66, *p* < 0.001.

**Conclusion:**

This study confirms that self-reported subjective cognitive impairment is common in breast cancer patients who received chemotherapy in a sample of South African patients. The results have implications for the overall care of cancer patients.

**Contribution:**

The context-based knowledge engendered by the current study is expected to augment the continuum of care for breast cancer patients.

## Introduction

Mounting evidence suggests that breast cancer is the most commonly diagnosed cancer amongst women worldwide (DeSantis et al. 2015), accounting for 11.6% of all cancers globally (Bray et al. 2018). Despite projections for a substantial increase in new cases, the survival rates of patients with breast cancer have shown an enormous improvement since 1989, and this is attributed to early detection of the disease and the availability of effective treatment modalities (Akram et al. 2017). For example, one study showed that the 5-year survival of breast cancer in 17 countries across the world increased to 85% (Allemani et al. 2018).

Globally, standard doses of an adjuvant chemotherapy regimen comprising of cyclophosphamide, methotrexate, fluorouracil (CMF) and that composed of fluorouracil, adriamycin, cyclophosphamide (FAC) have improved survival rates of breast cancer patients over time, especially in countries of low and middle income (Isakoff 2010; Verrill 2009). Current research across continents indicates that the therapeutic benefits derived from the use of chemotherapy for breast cancer have been accompanied by debilitating cognitive impairment (Loh et al. 2016; Weiss, Walker & Wiernik 2010). This condition (commonly referred to as ‘chemobrain’) manifests as diminished memory, concentration, attention and executive function (Ono et al. 2015; Simó et al. 2013) and its symptoms are detected in up to 75% of patients during chemotherapy. These symptoms can persist for years in up to 35% of these patients post-treatment (Janelsins et al. 2012; Vardy et al. 2008). Cancer, age, fatigue, anxiety, depression and hormonal therapy have been highlighted in research as confounding factors for cognitive impairment and they can diminish the patients’ quality of life (Munir et al. 2010; Selamat et al. 2014; Von Ah et al. 2013).

Most of the studies relating to chemobrain in patients with breast cancer were conducted amongst women in the United States of America (USA), Europe, Canada or Australia (Ribi 2012). This quantitative time-series thus sought to investigate the subjective cognitive function before, during and after chemotherapy in a group of female breast cancer patients in South Africa as measured on the Functional Assessment of Cancer Therapy-Cognition test (Fact-Cog Test). The significance of the study is that it will generate knowledge relevant to the local context with implication for the overall care and management of breast cancer patients.

## Materials and methods

### Research design

In a randomised, quantitative, time-series study, 30 out of 34 female patients with a breast cancer diagnosis prior to starting chemotherapy were included. Participants were aged 21–60 years at the time of recruitment. From the 34 patients, 4 were excluded (1 had previous chemotherapy exposure whilst 3 were over 60 years of age). All the participants were recruited from an outpatient oncology clinic at a semi-rural, tertiary hospital in Gauteng, during the period of October 2018 to October 2019 with the assistance of the treating oncologist, after confirming the stage of the patients’ breast cancer.

### Study measures

#### Participants’ health information

Patients diagnosed with stages II and III breast cancer who met inclusion criteria (not undergoing concurrent radiation therapy and hormonal therapy, non-fluency in English, no concurrent psychotic disorder, epilepsy and dementia) were considered eligible for inclusion in the study. After diagnosis, patients were assigned to receive either CMF or FAC according to the following schedules: CMF (cyclophosphamide 100 mg/m^2^ orally, methotrexate 40 mg/m^2^ intravenously, 5-fluorouracil 600 mg/m^2^ intravenously) every 3 weeks for six cycles and FAC (fluorouracil 500 mg/m^2^ intravenously, adriamycin 50 mg/m^2^ intravenously, cyclophosphamide 500 mg/m^2^ intravenously every 3 weeks for 6 cycles).

#### Cognitive assessment

The self-reported English version of the Fact-Cog (Version 3) was used to assess participants for chemobrain symptoms. The researcher tactfully assessed the participants’ English proficiency by randomly asking them a set of questions based on their comprehension of the participants’ information leaflet. The Fact-Cog instrument is a questionnaire with four subscales – perceived cognitive impairment (CogPCI), perceived cognitive ability (CogPCA), noticeability (CogOth) and impact on quality of life (CogQoL) – and it is used to measure each participant’s cognitive function throughout the course of chemotherapy (Park et al. 2019). This instrument includes 37 items that assess verbal fluency, concentration, memory, functional interference, mental acuity and multitasking ability (Vega et al. 2019). This study focused on the perceived cognitive impairment (range 0–72). A 5-point Likert scale (0 = never to 4 = several times a day) is used to rate each of the items over a 7-day period. Negatively and positively worded items are included in order to assess cognitive function. Higher scores on negatively worded statements indicate greater severity of cognitive impairment, whilst higher scores of positively worded ones indicate good cognitive function. On memory, for example, higher scores indicate better memory function. The Cronbach’s alpha (scale reliability) value for this subscale was 0.83 which demonstrates its acceptable reliability (Victorson et al. 2008).

### Procedure and data collection

Each participant was recruited with the assistance of the treating oncologist who made the diagnosis of breast cancer and determined the stages. The oncologist and/or the sister in-charge who were informed about the inclusion criteria of the study, assisted with the recruitment. After diagnosis and staging, either the oncologist or the sister-in-charge informed the patient about the study and explained what it involved. Those meeting the inclusion criteria were asked if they were interested in participating in the study. Patients who agreed were asked to wait in the waiting room in the breast cancer clinic, thereafter the researcher was introduced to the patient by the sister. The researcher verified that the patient met the inclusion criteria; thereafter he provided informed oral and written information about the study. Prior to receiving informed written consent and commencement of data collection, it was emphasised that the patient had the right to withdraw from the study at any point and/or refuse to participate in the study, without consequences for continued care at the clinic. Data collection involved completing a 5-min socio-demographic-health questionnaire at baseline, and a 10-min self-reported Fact-Cog measure at three points (baseline [T_0_], third cycle [T_1_], sixth cycle [T_2_]) during the participants’ scheduled visits to the breast clinic. Cognitive assessment of participants was done just after cancer staging and prior to commencement of chemotherapy (baseline [T_0_]), midway through chemotherapy (third cycle [T_1_]) and at the completion of chemotherapy (sixth cycle [T_2_]). Written informed consent was obtained from all the participants prior to the assessments.

### Ethical considerations

This study was approved by the Sefako Makgatho Health Sciences University Research and Ethics Committee (Protocol Number: /M/194/2018: PG) and the clinical director of Dr George Mukhari Academic Hospital. Participation in the study was voluntary and all patients had to provide informed consent. As part of the informed consent process, each of the participants was provided with detailed information about the study. It was also emphasised to them that they had the right to refuse to participate or to withdraw from the study without any consequences to their continued care. It was also clarified to each participant that the researcher does not form part of the treatment team and that information gathered during the study will be treated as confidential and will not be shared with others unless indicated by the participant. The participants were also informed that any personal information will be anonymised.

### Statistical analysis

Descriptive statistics (i.e. means, standard deviation [SD], etc.) were performed on the participant’s health and demographic information. The Fact-Cog scores for each treatment group were calculated. Pre-, during, and post-chemotherapy mean Fact-Cog scores were compared by paired *t*-tests and the non-parametric Wilcoxon signed-rank test. Differences between pre-, during and post-chemotherapy scores were analysed. All statistical analyses were performed on Statistical Analysis System (SAS Institute Inc., Carey, NC, USA), Release 9.4 or higher, running under Microsoft Windows for a personal computer. All tests were two-tailed and held statistical significance at *p* < 0.05.

## Results

### Participant characteristics

Of the participants, 33% (*n* = 10) were assigned to a chemotherapy regimen comprising of CMF, whilst 67% (*n* = 20) were treated with an FAC regimen. The mean age of the total participants was 50 years (*N* = 30), whilst that of the CMF group was 54 years and that of the FAC group was 48 years. The majority of the 30 participants had at least a high school educational qualification (18; 60%), whilst (19; 63%) were unemployed (see Table 1).

**TABLE 1:** Characteristics of participants.

Variable	CMF regimen	%	FAC regimen	%	*p*
**Mean age**	53.80	-	48.05	-	0.05*
**Age range**	-	-	-	-	0.12
31–50	02	20	11	55	
51–60	08	80	09	45	
**Ethnic group**					
Black African	10	33	20	67	
**Marital status**	-	-	-	-	-
Married	04	40	08	40	
Divorced	02	20	03	15	
Separated	00	00	01	05	
Widowed	01	10	05	25	
Single	03	30	03	15	
**Employment status**	-	-	-	-	-
Unemployed	07	70	12	60	
Full-time	02	20	02	10	
Part-time	01	10	06	30	
**Educational level**	-	-	-	-	0.15
Lower primary	00	00	00	00	
Primary	01	10	01	05	
Middle school	03	30	05	25	
High school	04	40	14	70	
Post school	02	20	00	00	
**Breast cancer stage**	-	-	-	-	1.00
II	04	40	08	40	
III	06	60	12	60	

CMF, Cyclophosphamide, methotrexate, fluorouracil; FAC, fluorouracil, adriamycin, cyclophosphamide.

Fisher exact tests.

* , Significance level set as *p* < 0.05.

### Chemotherapy-related subjective cognitive impairment

**FIGURE 1:**
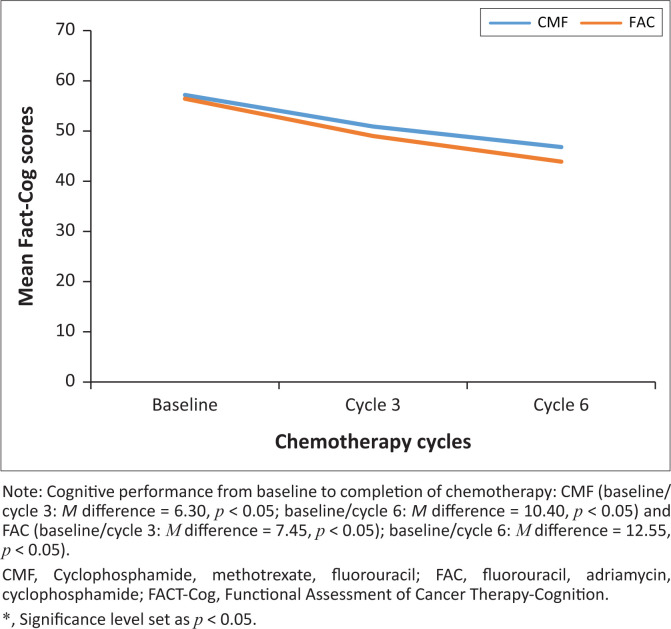
Perceived cognitive function and chemotherapy.

Of a total of 30 participants, 33% in the CMF group and 67% in the FAC group reported significant perceived cognitive impairment (*p* < 0.05). In the CMF group, there was significant change in the perceived cognitive function from baseline (*M* = 57.20, SD = 4.32) to T_2_ (cycle 6) (*M* = 46.80, SD = 12.00), *t* (9) = 2.91, *p* = 0.017 (see Table 2). Similarly, the FAC group demonstrated a significant decline in perceived cognitive function from baseline (*M* = 56.45, SD = 3.36) to T_2_ (cycle 6) (*M* = 43.90, SD = 12.88), *t* (19) = 4.66, *p* < 0.0002. The participants on the FAC regimen (*M* = 12.55, SD = 12.05) compared to the participants on the CMF regimen (*M* = 10.40, SD = 11.31) demonstrated greater perceived cognitive change from baseline to T_2_ (cycle 6) (see Table 2). The change was however not statistically significant (*t* [28] = 0.47, *p* = 0.642). Whilst these changes are not significant, they are still clinically meaningful, given that the FAC group showed greater perceived cognitive decline compared to the CMF group throughout the treatment (see Figure 1).

**TABLE 2:** Comparison of subjective self-reported cognitive function with chemotherapy regimens (cyclophosphamide, methotrexate, fluorouracil and fluorouracil, adriamycin, cyclophosphamide).

Variable	CMF					FAC				
	*N*	*M*	SD	*p*	*t*	*N*	*M*	SD	*p*	*t*
T_0_	10	57.20	4.32	< 0.0001	-	20	56.45	3.36	< 0.0001	-
T_1_	10	50.90	7.50	< 0.0001	-	20	49.00	7.61	< 0.0001	-
T_2_	10	46.80	12.00	< 0.0001	-	20	43.90	12.88	< 0.0001	-
T_0_ – T_1_	10	6.30	6.24	0.011*	3.19	20	7.45	6.90	0.0001*	4.83
T_0_ – T_2_	10	10.40	11.31	0.017*	2.91	20	12.55	12.05	0.0002*	4.66

Note: Differences in subjective cognitive function between chemotherapy regimens were compared using paired sample *t*-tests and Fisher exact tests.

CMF, Cyclophosphamide, methotrexate, fluorouracil; FAC, fluorouracil, adriamycin, cyclophosphamide; T_0_, baseline; T_1_, cycle 3; T_2_, cycle 6; SD, standard deviation.

* , Significance level set as *p* < 0.05.

## Discussion

The present study found that patients with breast cancer (stages II and III) had a decline in Fact-Cog scores from baseline (T_0_) to completion of chemotherapy (T_2_). These results of subjective cognitive complaints are consistent with previous studies which showed that patients with breast cancer are at high-risk for chemotherapy-related cognitive impairment (CRCI) (Jansen et al. 2011; Jim et al. 2012; Kohli et al. 2007). Noticeably, over the entire study, the patients showed a decline in Fact-Cog scores in both the CMF and FAC chemotherapy regimen groups. These results concur with existing research which showed that the administration of multiple cytotoxic agents such as CMF and FAC can lead to cognitive decline (Cheung, Chui & Chan 2012; Verrill 2009). These results are also consistent with evidence from a study that evaluated 42 patients with stage II and stage III breast cancer. The researchers found that in comparison to the non-chemotherapy group, the participants on chemotherapy (CMF and FAC) performed significantly worse on attention, memory and executive function tests (*p* < 0.05) (Chen et al. 2014).

Whilst both chemotherapy regimens were associated with ongoing cognitive decline in this study, the FAC group performed slightly worse than the CMF group on the Fact-Cog. Consistent with the current literature, anthracycline-based chemotherapy (adriamycin in the case of our study) exhibited a particularly high potential to induce cognitive decline (Kesler & Blayney 2016). This can potentially explain the results from our study. Other studies also highlighted increased cognitive impairment which was associated with the neurotoxic effects of the FAC regimen (Henderson 2015; Ramalho et al. 2017). Likewise, Schagen and Wefel (2013) found that chemotherapy induced-toxicity was associated with adverse short-term and long-term cognitive effects, based on both subjective-self reported and objective cognitive tests.

Whilst our study is in agreement with previous studies, what remain unclear are the mechanisms for the occurrence of CRCI. The perceived cognitive impairment related to chemotherapy appears to be subtle in some cases and similar results emerged in other studies when standard dose chemotherapy was administered to cancer patients (Pendergrass, Targum & Harrison 2018; Pereira et al. 2015). Perhaps the finding from the current study that FAC is associated with more cognitive problems as compared to CMF, could be explained in terms of anthracycline effects which have been associated with greater neuroinflammatory and neurotoxic consequences (Allen et al. 2019). Further, multicentre, prospective cohorts with a large longitudinal follow-up design to explore the effects of anthracycline-based regimens are needed.

### Limitations of the study

The study may have several potential limitations. Firstly, the small sample size could inhibit the generalisation of the findings around the effect of chemotherapy on subjective cognitive function of patients with breast cancer. Secondly, we only assessed subjective cognitive impairment and did not administer a battery of objective neuropsychological testing. Thirdly, in this study, only one subscale, which is cognition perceived cognitive impairment, was used and therefore aspects related to quality of life were excluded. Furthermore, the focus of this study was not to explore the underlying mechanism that can explain the observed cognitive decline in patients undergoing chemotherapy.

## Conclusion

Although more work, which includes objective neurocognitive testing of larger samples of breast cancer patients, is needed to establish the full extent of cognitive change during and following chemotherapy, our preliminary results do suggest ongoing cognitive problems associated with chemotherapy in a small sample of women with stage II and stage III breast cancer, irrespective of the chemotherapy regimen. The perceived cognitive impairment observed in this study provides clinically meaningful information for the management of patients as greater perceived cognitive decline was demonstrated by the FAC group compared to the CMF group and this could assist clinicians to choose chemotherapy regimens with tolerable effects for their patients. Whilst cognitive deficits may be associated with chemotherapy-treated breast cancer patients and may impact on day-to-day functioning, it is not necessarily apparent. The study may, nevertheless, contribute to the documenting of the reality of chemobrain locally.
